# Microglia activation in sepsis: a case-control study

**DOI:** 10.1186/1742-2094-4-4

**Published:** 2007-01-15

**Authors:** Afina W Lemstra, Jacqueline CM Groen in't Woud, Jeroen JM Hoozemans, Elise S van Haastert, Annemiek JM Rozemuller, Piet Eikelenboom, Willem A van Gool

**Affiliations:** 1 Department of Neurology, Academic Medical Centre, PO Box 22660, 1100DDAmsterdam, The Netherlands; 2 Department of Neuropathology, Academic Medical Centre, PO Box 22660, 1100DDAmsterdam, The Netherlands

## Abstract

**Background:**

infection induces an acute phase response that is accompanied by non-specific symptoms collectively named sickness behavior. Recent observations suggest that microglial cells play a role in mediating behavioral changes in systemic infections. In animal models for sepsis it has been shown that after inducing lipopolysaccharide, LPS, microglia in the brain were activated. The aim of this study was to investigate whether activation of microglia can be detected in patients who died of sepsis.

**Methods:**

in a case-control study brain tissue of 13 patients who died with sepsis was compared with that of 17 controls. Activated microglia were identified by expression of MHC-class II antigens and CD68. Microglia activation was analyzed by a semiquantitative score combining both the number of the immunoreactive cells and their morphology.

**Results:**

in patients who died with sepsis there was a significant increase in activated microglia in the grey matter when stained with CD68 compared to controls. This effect was independent of the effect of age.

**Conclusion:**

this study shows for the first time in human brain tissue an association between a systemic infection and activation of microglia in the brain. Activated microglia during sepsis could play a role in behavioral changes associated with systemic infection.

## Background

Infection by pathogenic microorganisms triggers an acute phase response which manifests itself with fever, neuro-endocrine changes and behavioral changes. The non-specific symptoms accompanying severe infection such as malaise, fatigue, anorexia, hypo-and hypersomnia, depression and lethargy, are collectively referred to as sickness behavior[1,2].

The pathophysiological mechanisms underlying sickness behavior remain to be elucidated. Several studies suggest that pro-inflammatory cytokines such as tumor necrosis factor α (TNF-α), interleukin (IL)-1 and IL-6 induce these responses[3,4]. Experiments in both animals and humans showed that administering cytokines and lipopolysaccharide (LPS, the active fragment of Gram-negative bacteria) peripherally or directly in the brain, causes cognitive impairments and behavioral disturbances[2,5-9]. Some observations suggest that microglial cells play a role in mediating behavioral changes in systemic infections. Microglia are considered the macrophages of the brain which can be triggered from a resting state into an activated state by exogenous stimuli. Activation in this context means that microglia change their morphology and upregulate and express antigens. Semmler et al. showed that in rats a peripheral inflammatory reaction induced by LPS, as a model for sepsis, lead to profound activation of glial cells in the brain, including microglia[10]. Mattiace et al described upregulation of cell surface antigen on microglia in patients with Alzheimer's disease and carcinomatosis with concomitant infection[11].

We analyzed in a case-control study the distribution of immunophenotype of microglia in brain tissue from patients with sepsis, defined as the presence of both microbiologically confirmed infection and a systemic inflammatory response. In this study we present the first evidence for microglia activation in the human brain associated solely with a systemic inflammatory reaction.

## Materials and methods

### Selection of cases and controls

Data were obtained of all brain autopsies that had been performed on patients who died in the Amsterdam Medical Centre (AMC) in the period of the year 2001 to the year 2005. In total the autopsy reports of 170 patients were studied.

**Figure 1:**
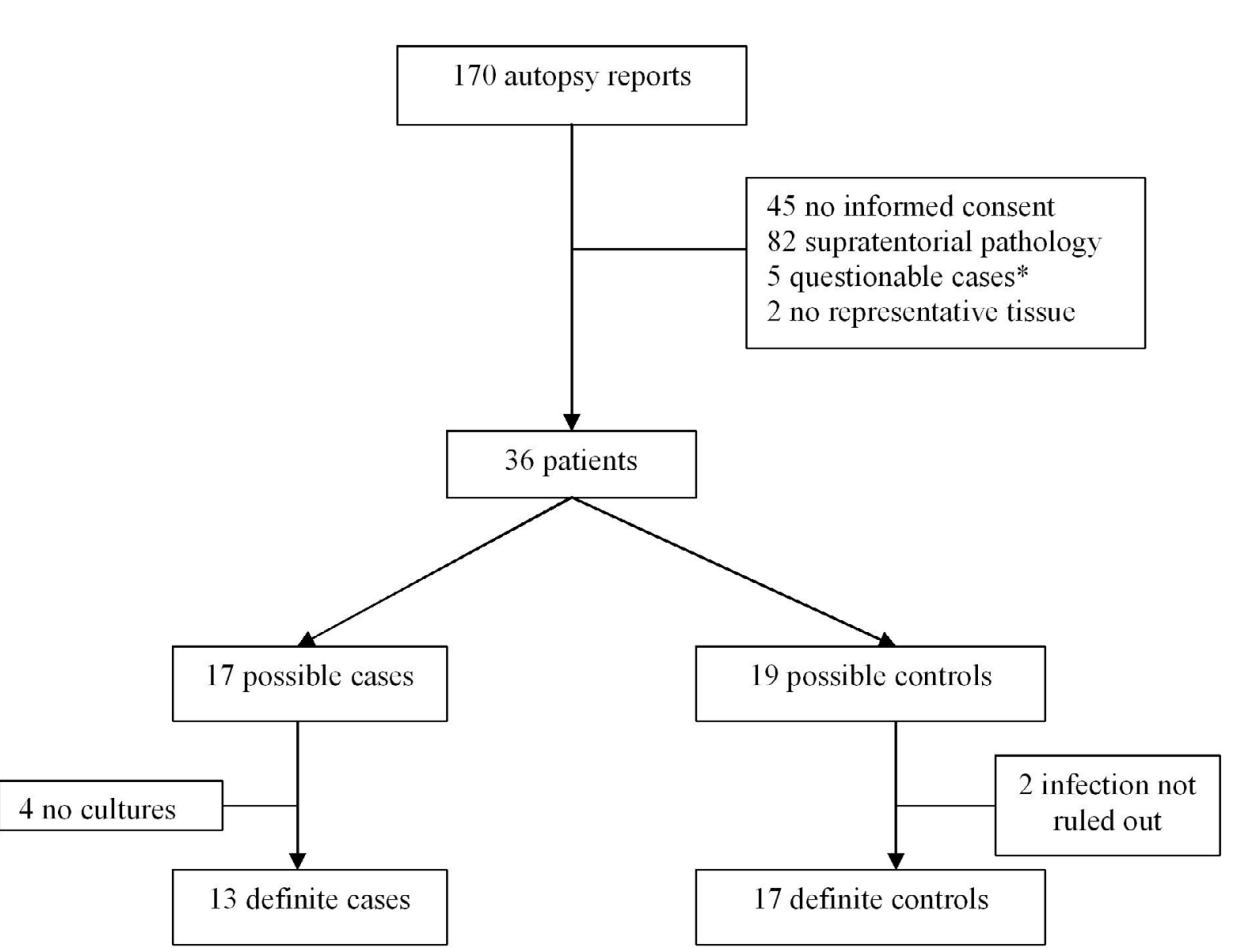
**Flow chart of selection of cases and controls.** * Questionable cases: systemic diseases or diseases with a (possible) effect on the central nervous system/brain (HIV-infection, non-Hodgkin lymphoma, lungcancer)

Patients were selected by the following criteria: permission for use of autopsy material for research purposes, presence of frontal and parietal brain tissue and absence of signs of CNS infection and supratentorial pathology including gliosis and senile changes (Figure 1).

Thirty-six patients fulfilled these criteria and were subsequently screened for having sepsis at the time of death. A patient was considered to have died with sepsis if blood cultures were positive up to 7 days before death or if post-mortem cultures (spleen, kidney or lung) were positive. Supporting evidence for sepsis was collected from medical records and computerised laboratory charts. This comprised clinical signs as fever and tachycardia and laboratory findings such as leucocytosis and elevated CRP according to the international consensus criteria for the definition of sepsis[12]. In total 13 patients were selected as cases.

Patients were classified as control if sepsis at time of death was highly unlikely. This was determined by negative cultures (saliva, urine, blood, faeces and post-mortem), normal CRP and white blood cell count 0 to 3 days before death and/or no signs of infection in the autopsy report. In total 17 patients were considered suitable controls. The predominant cause of death in controls was of cardiac or circulatory origin (Table 1). From all patients brain tissue from frontal and/or parietal regions was derived and used for analysis.

**Table 1:** Cause of death in controls

*Pt.nr*	*Cause of death*
1	Arrhythmia
2	Cardiac failure
3	Dying heart (trauma)
4	Brain herniation due to Arnold-Chiari malformation
5	Acute heartfailure
6	Myocardial infarction
7	Cardiac failure
8	Haemorrhagic shock
9	Myocardial infarction
10	Haemorrhagic shock
11	Haemorrhagic shock
12	Euthanasia (endometrial carcinoma)
13	Cardiopulmonary insufficiency
14	Haemorrhagic shock
15	Myocardial infarction
16	Myocardial infarction
17	Haemorrhagic shock

### Immunocytochemistry/immunohistochemistry

For the immunohistochemical stainings 4% formalin fixed, paraffin-embedded tissue from the neocortex was used. Brain tissue samples were selected that contained frontal and/or parietal cortex and the adjacent sub-cortical white matter. Sections (7 μm) were mounted on APES coated (frost plus) tissue slides and deparaffinised. Subsequently sections were immersed in 0.3% H_2_O_2 _in methanol for 30 minutes to quench endogenous peroxidase activity. For antigen retrieval, sections were pretreated in 10 mM pH 6.0 citrate buffer and heated by autoclave for 10 minutes. Serum and antibodies were dissolved in phosphate-buffered saline (PBS). Sections were pre-incubated for 30 minutes with 10% normal goat-serum (DAKO, Glostrup, Denmark) and subsequently incubated with the primary antibodies (4°C overnight). As primary antibodies anti-Human Leukocyte Antigen (HLA)-DP, -DQ, -DR (mouse monoclonal, clone CR3/43, DAKO; 1:100) and anti-CD68 (mouse monoclonal, clone PG-M1, DAKO, 1: 200) were used. For the detection of mouse antibodies ready-for-use Power Vision (ImmunoLogic, Duiven, the Netherlands) was used according to the instructions of the manufacturer. Before use Powervision solutions were diluted 1:1 in PBS. Color was developed using 3,3'-diaminobenzidine (0.1 mg/ml, 0,02% H_2_O_2_, 3 min.) as chromogen and nuclei were stained with haematoxylin. Sections were mounted with Entellan (Merck, Darmstadt, Germany).

### Evaluation of immunostaining

**Figure 2:**
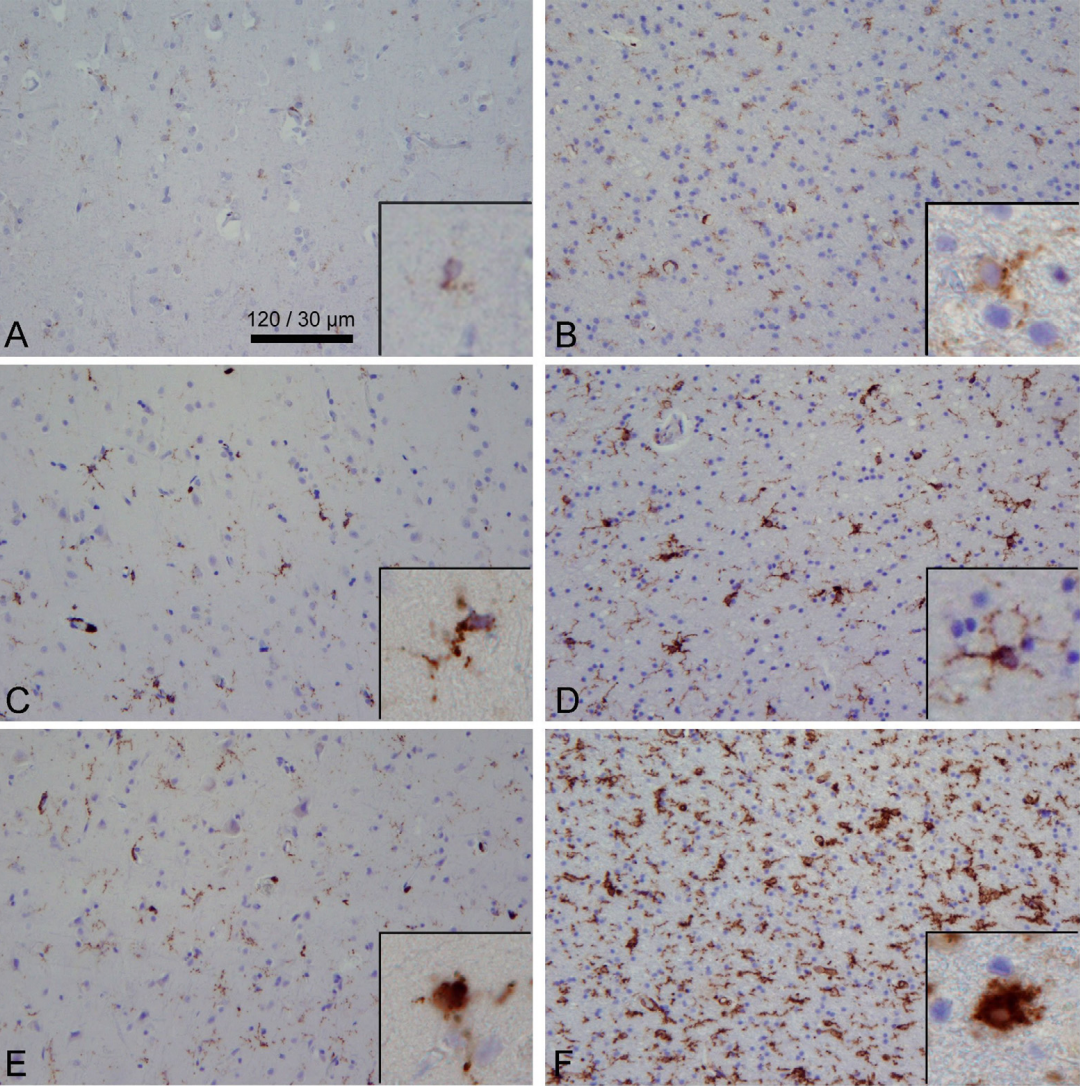
**Immunohistochemical detection and immunoscore classification**; left column shows CD68 grey matter (A, C & E) and right column shows MHC-class II in white matter (B, D, & F); top is score 1, middle is score 2 & bottom is score 3 (*see Materials & Method section*). Shown are ×40 magnification microscopic fields of representative immunohistochemical stainings; all pictures are from sepsis patients, except for C. Insets show the morphological change of microglia.

A semi-quantitative assessment of microglial immunoreactivity in the parenchyma for CD68 and MHC-class II antigens was carried out. All sections were evaluated independently by three observers blinded for case number and clinical information. One representative section was assessed for each patient at ×20 magnification after which a 3-field count was performed at ×40 magnification. Cortical fields were selected so that all cortical layers were included. White matter fields included both "sub grey matter" as well as deep white matter. Immunostainings for CD68 and MHC-class II antigens were rated on a three point scale. For the rating both the number of immunopositive cells and morphological changes were taken into account. Grey matter and white matter were rated independently. For the grey matter the following scoring definitions were applied: score 1 = less then 5 immunoreactive cells; score 2 = between 5 and 15 immunoreactive cells of which less than 25% with an amoeboid morphology; score 3 = more than 15 immunoreactive cells of which more than 25% have an amoeboid morphology. For the white matter the following definitions were applied: score 1 = less than 10 immunoreactive cells; score 2 = between 10 and 25 immunoreactive cells of which less than 25% with an amoeboid morphology; score 3 = more than 25 immunoreactive cells of which more than 25% have an amoeboid morphology. For score 1 no notice of amoeboid cells was made. For score 3 each section had to fulfill both criteria: more than 15 (grey matter) or 25 (white matter) immunoreactive cells and more than 25% amoeboid morphology. Sections only meeting one of these criteria were classified as score 2. Figure 2 shows representative pictures of the different immunoreactive scores.

In sections with area variations in the density of immunoreactive cells in the studied specimen an average score was given after reaching consensus between the three observers. Perivascular macrophages were noted separately but not included in the final evaluation since parenchymal changes were the object of interest. Some sections showed immunoreactive staining of endothelia as well as appearance of small clusters (40–60 μm) of microglia in the grey matter. These were noted separately but not included in the semi-quantitative score.

### Statistics

SPSS for Windows was used for statistical analysis of the data. Mann-Whitney U test for non-parametric testing was used to assess differences between groups.

Because of the nature of the outcome variable (i.e. microglia activation score) an ordinal regression model was used to predict effects of independent variables available through SPSS procedure PLUM (polytomous logit universal models).

A value of *P *< 0.05 was considered statistically significant.

## Results

### Cases and controls

Demographic characteristics of cases and controls are listed in Table 2. There were no differences in sex and in time from death until autopsy between cases and controls. There was a difference of 9.3 years in the mean age between the two groups, of which the controls were younger.

**Table 2:** Patients characteristics

	*Cases*	*Controls*
**Number**	13	17
**Age (years)**	71.2 (37 – 92)	61.9 (40 – 85)
**Sex (male : female)**	6 : 7	8 : 9
**Post mortem time (hours)**	23.6 (6.0 – 63.0)	29.5 (7.0 – 77.0)

### Qualitative analysis of the immunostaining

Cells of the microglia/macrophage cell system were immunohistochemically identified in both cases and controls. Microglia cells were diffusely distributed throughout the parenchyma. In agreement with other reports the number of microglia was in general higher (about two times) in the white matter than in the cortex. Immunoreactivity was also observed in the perivascular space but was not taken into account for further quantitative analysis. Next to microglia, endothelial cells stained positive in 2 cases and in 2 controls. Some clustering of microglia was observed in a few specimen in both cases and controls.

### Semi-quantitative analysis of the distribution of immunoreactive parenchymal cells

**Figure 3:**
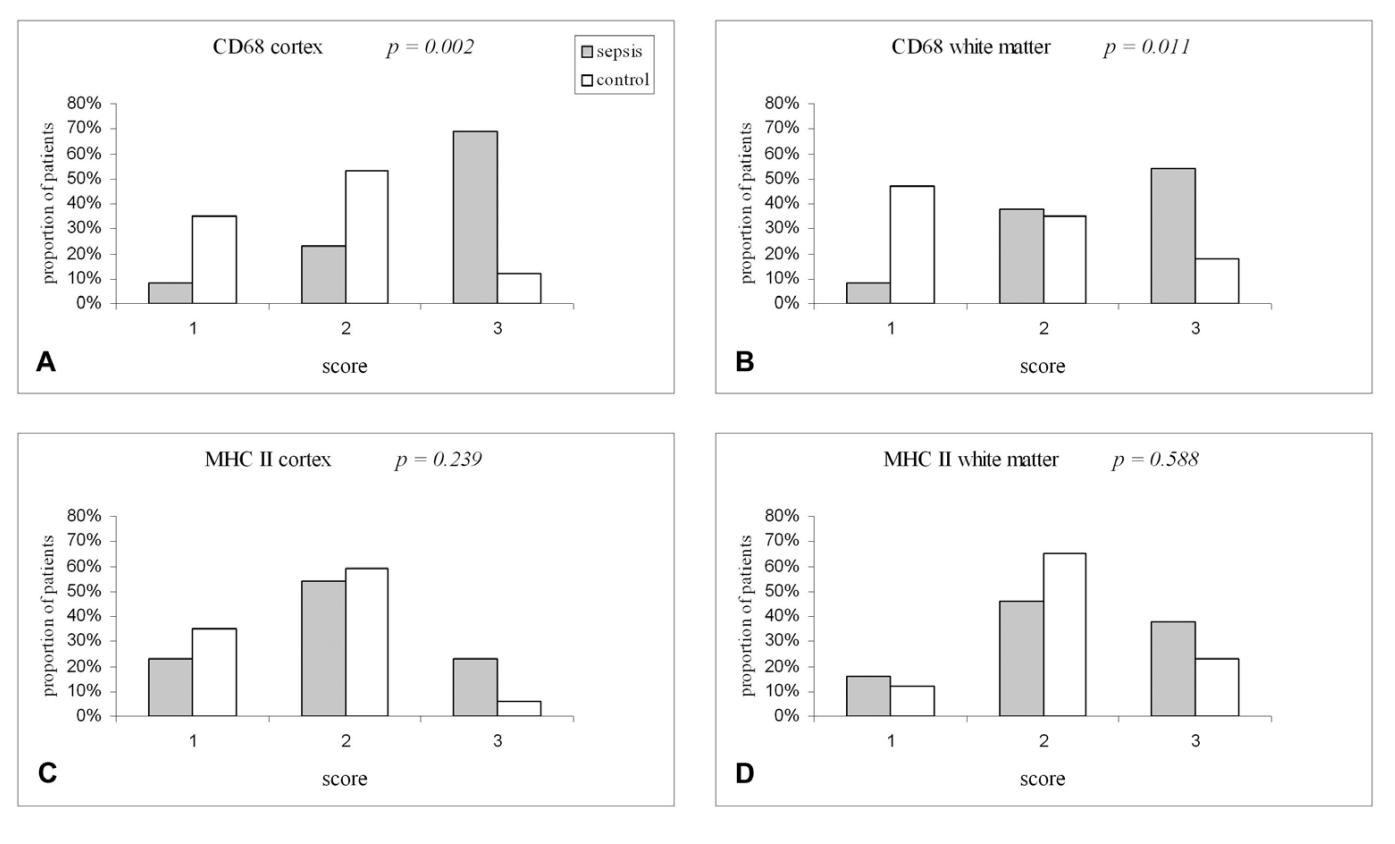
Distribution of microglial activation scores in septic patients and controls in cortex and white matter after immunohistochemical staining for CD68 (A, B) and MCH-class II antigens (C, D).

In 9 of the 13 sepsis patients (69%) a diffuse distribution of CD68 immunoreactivity with a high number of positive cells with an amoeboid (or macrophage-like) appearance was observed in the cortex. Only 1 case (8%) showed a low number of microglia that were mainly in the resting state (score 1) compared to 6 of the controls (35%). The differences in the proportion of CD68 positive cells between the cases and controls in the CD68 immunostaining were significant both in the cortex (p = 0.002) and in the white parenchyma (p = 0.011) (Fig 3A&B).

The typical staining pattern for the MHC-class II antigens was clearly seen: the contour of the microglia was stained which made the cell processes more visible. In contrast to the results of the CD68 staining, there was no significant difference between cases and controls in the proportions of MHC-class II immunoreactive cells: cortex p = 0.239, white parenchyma p = 0.588 (Fig 3C&D).

### Microglia activation and age

In order to investigate if the difference in mean age between our cases and controls could explain the observed differences in CD68 immunostaining we did an ordinal regression analysis with the level of microglial activation as dependent variable, group as factor and age as covariate. In this model having sepsis had a significant and independent effect on microglia activation when CD68 positive cells were observed in both grey and white matter (p = 0.009 and p = 0.013 resp.). Age also had a significant effect on microglia activation when stained with CD68 but only in grey matter (p = 0.021). The parameter estimates of respectively age and having sepsis were 0.069 and 2.329 in the model for CD68 in grey matter.

## Discussion

In animal studies it has been shown that when sepsis is experimentally induced by LPS injection, microglia in the brain become activated[10]. Our aim was to replicate this finding in human tissue. In this study we describe for the first time a marked increase of activated microglia in brain tissue of patients who died with sepsis. This case-control study was conducted in an unbiased autopsy sample which adds to the external validity of our results. We used the antibody CD68 and MHC-class II antigens which are widely used markers for microglia activation. In addition to immunophenotype, the morphology of the microglia was taken into account for the microglia activation score. The CD68 expression on microglia was significantly higher in the septic patients compared with the controls and more amoeboid formed microglia were seen. We did not find a significant difference between sepsis patients and controls when MHC-class II immunoreactivity was observed.

There was a difference of 9.3 years in the mean age of the sepsis-group compared to the control-group. This could have influenced our results. When we investigated the effects of sepsis and age in an ordinal regression model for CD68 immunoreactivity there was a independent and significant effect of sepsis on microglia activation next to the effect of age.

In the present study the mean age in *both *cases and controls was fairly high. This could explain why we found a discrepancy between the results of the CD68 and the MHC-class II immunoreactivity. CD68 is a lysosomal membrane marker and stains microglia in a highly active, phagocytising state [13-15]. HLA-DP,-DQ,-DR is an antibody directed against MHC-class II antigens and stains microglial cells with great morphologic heterogeneity, irrespective of the level of activation[16].

Microglia usually remain in a ramified resting form but age may alter this quiescent state as is seen by an upregulated MHC-II expression [17-19]. It has already been suggested that MCH-II expression is a marker of cell aging in addition or even instead of being an activation marker [19,20]. Since our study population is relatively old, MHC-II staining could partly reflect also microglial senescence and therefore not yield significant differences between sepsis and controls, due to a ceiling effect. Our results could reflect an exaggerated microglial activation caused by sepsis, as is shown by CD68-immunostaining, in addition to the effect of aging which is expressed by the MHC-class II immunoreactivity.

Several limitations are connected to this study. Data were collected retrospectively; therefore sepsis in the cases as well as the absence of a systemic inflammatory response in controls could not be uniformly defined in all patients as could have been done in a prospective study. There were differences in the time that elapsed between cultures and death of the patients and in some patients more clinical data would have been helpful. Technical factors like tissue handling and post-mortem delay can affect the detection of monocyte/macrophage receptors as shown for MHC-class II expression[11]. In our study, mean time from death until autopsy did not differ between both groups and post-mortem tissue preparation was equal in both groups since this was done in the same laboratory using one protocol.

Our findings concur with the accumulating evidence from experimental studies that the brain actively participates in systemic infections. It is acknowledged that the peripheral immune system has a profound impact on the brain. Several pathways have been proposed that may play a role in this cross-talk, but the activation of microglia in the brain parenchyma seems to be pivotal[21,22]. Animal studies, in which LPS was administrated as a model for sepsis, revealed microglia activation and cytokine release in cerebral tissue, thus showing the ability of the central nervous system to induce an innate immune response when triggered by pathogens[7,23]. Our study provides the first evidence of an immunological response, i.e. activated microglia, in *human *brain tissue related to a systemic inflammatory reaction.

Highly active microglia may be involved in sickness behavior observed in patients with severe systemic infection. Sickness behaviour is thought to be a highly conserved reaction of the body and part of our normal homeostasis. These symptoms are usually reversible if the underlying cause is treated properly[24].

It has been demonstrated in mice that activation of the peripheral innate immune system by intraperitoneal injection of lipopolysaccharide (LPS) next to microglia activation and cytokine release is associated with behavioural deficits[3,7,22]. In healthy volunteers injection of low-dose endotoxin caused increasing levels of pro-inflammatory circulating cytokines. This was associated with memory disturbances and behavioural changes[5,8]. Circulating cytokines in a systemic inflammatory response seem to be able to signal the brain and modify the cytokine profile within specific CNS regions[2,6]. A heightened neuroinflammatory response and a modified cytokine milieu may lead to neurobehavioral impairments and even delirium. These consequences are even more distinct in animals with certain pre-existing conditions such as high age or chronic neurodegeneration in which microglia are already "primed"[7,24,25]. Deng et al showed that in old rats compared to young and middle aged rats there is an enhanced activation of microglia when cytokines were injected into the brain[26]. It is tempting to speculate that similar mechanisms may play a role in humans, since both old age and neurodegenerative diseases predispose for delirium. Research to evaluate the production of cytokines like IL-1, IL-6 and TNF-α by activated microglia in septic patients might strengthen the hypothesis of the role of microglia in sepsis and co-existing neurological symptoms.

Our study is a first step in testing this hypothesis by showing an actual increase in activated microglia in patients who died of sepsis.

## Competing interests

The authors declare that they have no competing interests.

## Authors' contributions

AWL, AJMR, PE and WAG designed the study. AWL coordinated the study and was responsible for writing the manuscript. JCMG carried out most of the lab work and analysed the data. JJMH rated the sections and participated in writing the manuscript. ESH rated the sections and assisted with the lab work. AJMR was responsible for the autopsy material. All authors read and approved the final manuscript.
